# Nerve Agent Hydrolysis Activity Designed into a Human Drug Metabolism Enzyme

**DOI:** 10.1371/journal.pone.0017441

**Published:** 2011-03-18

**Authors:** Andrew C. Hemmert, Tamara C. Otto, Roberto A. Chica, Monika Wierdl, Jonathan S. Edwards, Steven L. Lewis, Carol C. Edwards, Lyudmila Tsurkan, C. Linn Cadieux, Shane A. Kasten, John R. Cashman, Stephen L. Mayo, Philip M. Potter, Douglas M. Cerasoli, Matthew R. Redinbo

**Affiliations:** 1 Department of Biochemistry/Biophysics and Chemistry, University of North Carolina at Chapel Hill, Chapel Hill, North Carolina, United States of America; 2 United States Army Medical Research Institute for Chemical Defense, Aberdeen Proving Ground, Maryland, United States of America; 3 Department of Biology and Chemistry, California Institute of Technology, Pasadena, California, United States of America; 4 Department of Chemical Biology and Therapeutics, St. Jude Children's Research Hospital, Memphis, Tennessee, United States of America; 5 Human BioMolecular Research Institute, San Diego, California, United States of America; University Paris Diderot-Paris 7, France

## Abstract

Organophosphorus (OP) nerve agents are potent suicide inhibitors of the essential neurotransmitter-regulating enzyme acetylcholinesterase. Due to their acute toxicity, there is significant interest in developing effective countermeasures to OP poisoning. Here we impart nerve agent hydrolysis activity into the human drug metabolism enzyme carboxylesterase 1. Using crystal structures of the target enzyme in complex with nerve agent as a guide, a pair of histidine and glutamic acid residues were designed proximal to the enzyme's native catalytic triad. The resultant variant protein demonstrated significantly increased rates of reactivation following exposure to sarin, soman, and cyclosarin. Importantly, the addition of these residues did not alter the high affinity binding of nerve agents to this protein. Thus, using two amino acid substitutions, a novel enzyme was created that efficiently converted a group of hemisubstrates, compounds that can start but not complete a reaction cycle, into *bona fide* substrates. Such approaches may lead to novel countermeasures for nerve agent poisoning.

## Introduction

**Figure 1:**
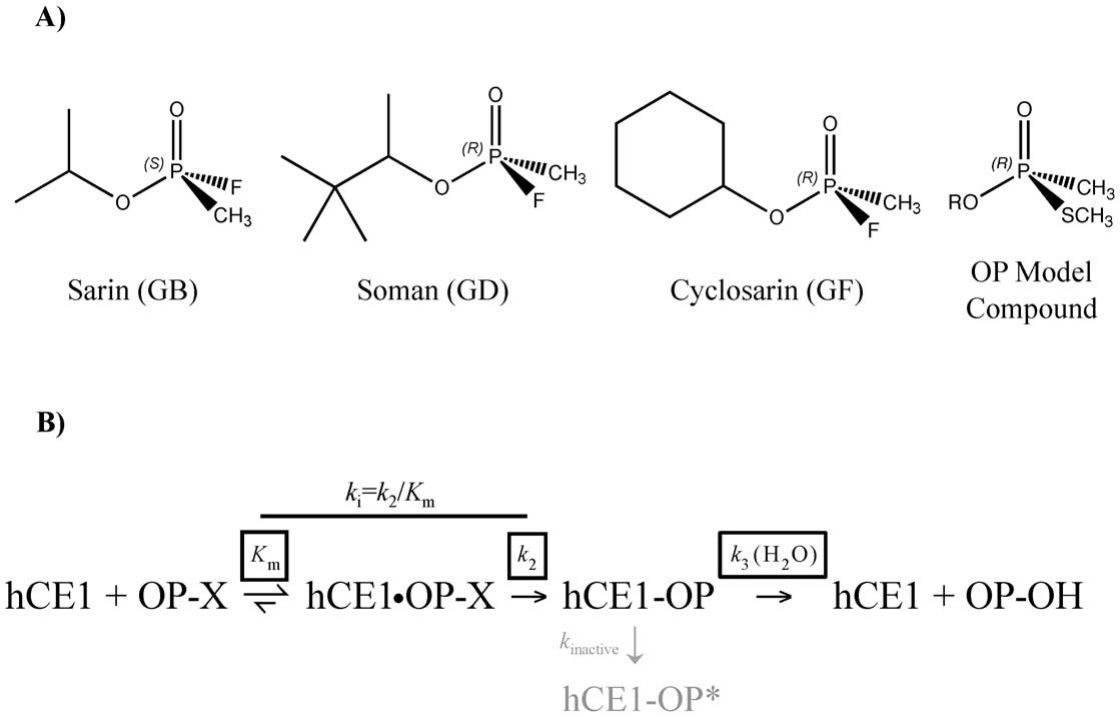
Organophosphate (OP) inhibition of human carboxylesterase 1 (hCE1). **A**. Three G-type OP nerve agents and OP model compound (R represents respective *O*-alkoxy groups). Wild-type hCE1 preferentially binds the stereoisomers shown (7). **B**. Schematic mechanism of OP hydrolysis by hCE1. X represents the leaving group and * denotes a non-reactive state.

Organophosphorus (OP) nerve agents are some of the most poisonous chemicals known (**Figure 1A**) [1]. By covalently phosphonylating the catalytic serine of the neurotransmitter-regulating enzyme acetylcholinesterase (AChE), OPs produce a cholinergic crisis that can cause respiratory failure and death [2]. OP nerve agents have been used as chemical weapons by both established governments and terrorist groups [2], [3]. In addition, there are an estimated 250,000 deaths worldwide each year associated with OP pesticides [4]. Thus, there is significant interest in developing novel approaches to detoxify these compounds for military, security and clinical applications [5].

Human carboxylesterase 1 (hCE1) is primarily expressed in the liver and metabolizes a variety of chemically distinct substrates, from potentially harmful endogenous compounds to exogenous compounds including drugs and environmental toxins [6]. hCE1 is homologous in structure and catalytic mechanism to AChE and exhibits features that make it attractive as a potential nerve agent hydrolase [6]. Both hCE1 and AChE employ a catalytic triad consisting of serine, histidine, and glutamic acid residues. In addition, for both enzymes, OP nerve agents act as hemisubstrates – compounds that can start but not complete a reaction cycle to regenerate free active enzyme [7], [8], [9]. Indeed, hCE1 is potently inhibited by nerve agents, forming long-lived covalent phosphonyl-enzyme complexes (**Figure 1B**) [8]. We have previously shown that hCE1 does not undergo the dead-end “aging” process from this intermediate that is observed for the cholinesterases, AChE or butyrylcholinesterase (BChE) [10]. In addition, wild-type (wt) hCE1 exhibits a slow hydrolysis-based reactivation after exposure to sarin, but no reactivation is observed when hCE1 is exposed to soman or cyclosarin [8].

Thus, we sought to design an hCE1 variant capable of hydrolyzing OP nerve agents. We employed molecular modeling techniques based on crystal structures of hCE1 in trapped covalent complexes with agents to engineer amino acids capable of promoting dephosphonylation. Herein, we report the placement of a pair of residues in the hCE1 active site that significantly increases the rate of nerve agent hydrolysis relative to native hCE1, while still retaining high affinity and selective functional substrate activity towards these compounds. Thus, using only two amino acid changes, we have created one of the most efficient enzymes engineered to date with respect to converting hemisubstrates into substrates.

## Results

### Structure-Guided Dyad Design

**Figure 2:**
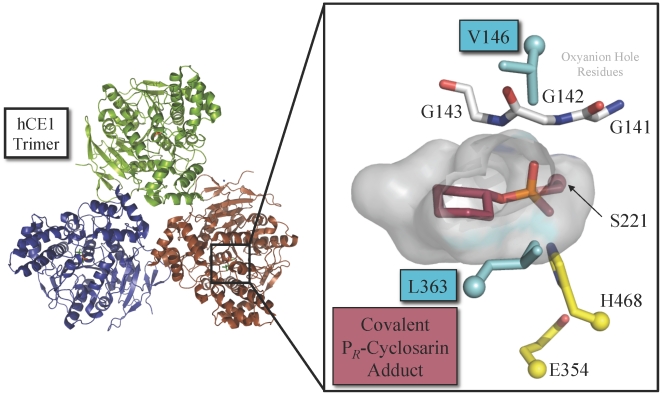
Human carboxylesterase 1 active site structure. Active site of human carboxylesterase 1 covalently inhibited via S221 with cyclosarin (magenta) [8]. The other catalytic residues, in addition to S221, are H468 and E354 (yellow), and are surrounded by hydrophobic residues (grey surface) including V146 and L363 (light blue), as well as the oxyanion hole (white).

To hydrolyze the covalent phosphonyl-enzyme OP-bound species of hCE1, we introduced a histidine (H) at position 146 and a glutamic acid (E) at position 363. These residues were chosen because they appeared ideally positioned to order a water molecule for hydrolysis. The corresponding wild-type residues V146 and L363 are positioned on either side of the active site pocket, adjacent to the OP-serine bond formed during the two-step catalytic mechanism of the enzyme (**Figure 2**). V146 is part of an extended loop between β7 and α2 that also contains the oxyanion hole formed by residues G142 and G143. L363 caps α12, at the edge of the hCE1 active site. As outlined below, the structurally similar residue glutamine (Q) was used as a control mutation for each position. hCE1 mutants were successfully expressed, purified, and shown to exhibit enzyme activity using a standard esterase substrate para-nitrophenylbutyrate (pNPB) [11], although wildtype hCE1 was ∼30-fold faster toward pNPB than the V146H/L363E mutant (660 nmol/min/mg vs. 21 nmol/min/mg).

### Enhanced Nerve Agent Hydrolysis

**Figure 3:**
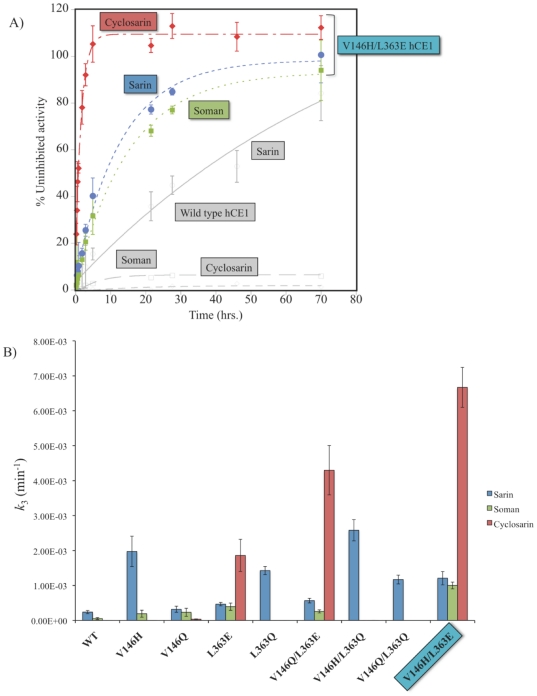
Reactivation of hCE1 following nerve agent exposure. **A**. Spontaneous reactivation of V146H/L363E hCE1 following inhibition by racemic sarin (blue), soman (green), or cyclosarin (red). Wild type hCE1 (grey) only reactivates following sarin inhibition (7). n = 6, s.d. **B**. Rates of dephosphonylation for hCE1 variants in the presence of sarin (blue), soman (green) and cyclosarin (red). n = 3, s.d.

The V146H/L363E hCE1 mutant exhibited a significant increase in enzyme reactivation following nerve agent inhibition (**Figure 3A**). We had shown previously that wild-type (wt) hCE1 reactivates following sarin exposure at a rate (*k*_3_) of 2.4±0.50×10^−4^ min^−1^, or a half-time of reactivation (t_1/2_) of 46 hrs, and that wt enzyme was not able to recover following soman or cyclosarin inhibition [8]. In contrast, however, the V146H/L363E hCE1 mutant spontaneously reactivated following sarin and soman inhibition with rates of 12±2.0×10^−4^ min^−1^ (t_1/2_ of 9.5 hrs) and 10±1.0×10^−4^ min^−1^ (t_1/2_ of 11.5 hrs), respectively. The reactivation rates of V146H/L363E hCE1 after sarin and soman inhibition were 5- and 20-fold faster, respectively, than the wt enzyme. Most strikingly, the designed V146H/L363E dyad exhibited a rate of reactivation of 67±6.0×10^−4^ min^−1^ (t_1/2_ of 1.2 hr) following cyclosarin exposure, an increase in reactivation of ∼33,000-fold relative to wt enzyme (where the wt rate was estimated to be 1×10^−5^ min^−1^, the lower limit of rate measurement). Recall that no reactivation was observed with wt hCE1 in the presence of this OP. Thus, efficient recovery of carboxylesterase activity following nerve agent exposure was achieved by introducing a pair of residues adjacent to the hCE1 active site.

We next tested a series of single and double mutant controls containing Q residues at positions V146 and L363 to examine the effect of each mutant on reactivation rate (*k*_3_) with respect to sarin, cyclosarin, and soman hydrolysis (**Figure 3B** **,** **Table 1**). For sarin hydrolysis, introduction of a polar side chain (H or Q) in either position alone or in combination produced similar rates of reactivation, ranging from 3.2×10^−4^ to 26×10^−4^ min^−1^, up to a 11-fold increase relative to wt hCE1. Following soman or cyclosarin inhibition, L363E appeared critical to increasing *k*_3_ from sub-detection limits in wt hCE1 to 4.0×10^−4^ and 19×10^−4^ min^−1^, up 8- and 9,300-fold, respectively (**Table 1**). For soman, polar residues at position 146 (H or Q) weakly stimulated reactivation; however, when V146H was combined with L363E, reactivation increased 20-fold relative to wt hCE1 (**Table 1**).

**Table 1:** Rates of dephosphonylation for hCE1 mutants.

hCE1	Sarin		Soman		Cyclosarin	
	*k*_3_ (×10^−4^ min^−1^)	Fold Increase	*k*_3_ (×10^−4^ min^−1^)	Fold Increase	*k*_3_ (×10^−4^ min^−1^)	Fold Increase*
WT	2.4±0.5	-	0.5±0.3	-	<0.01	-
V146H	20±4	8	2±1	4	0.032±0.006	16
V146Q	3.2±0.9	1	2±1	4	0.34±0.09	171
L363E	4.6±0.5	2	4±1	8	19±5	9,300
L363Q	14±1	6	<0.01	-	<0.01	-
V146Q/L363E	5.7±0.7	2	2.6±0.5	5	43±7	21,500
V146H/L363Q	26±3	11	<0.01	-	<0.01	-
V146Q/L363Q	12±1	5	<0.01	-	<0.01	-
V146H/L363E	12±2	5	10±1	20	67±6	33,355

N.R. is no reactivation with a detection limit <1×10^−5^ min^−1^.

pH 7.4, 25°C, N = 3, s.d.

*k_3_∼1×10^−7^ min^−1^, i.e. (1×10^−5^ min^−1^)*(1% activity)/100.

However, the largest *k*_3_ enhancement was for cyclosarin and the V146H/L363E mutant. This designed enzyme exhibited an increased rate of enzyme-mediated cyclosarin dephosphonylation of >33,000-fold relative to the wt enzyme (**Table 1**). A second double-mutant in which a Q at position 146 was combined with an E at position 363 was also found to exhibit robust enzyme reactivation following cyclosarin exposure, with an rate increase of >21,000-fold relative to wt hCE1 (**Table 1**). Thus, a glutamic acid at position 363 appeared to function as a catalytic base to enhance cyclosarin reactivation; a glutamine in that position failed to stimulate enzyme activity in the presence of cyclosarin. Taken together, these data demonstrate that targeted mutagenesis within hCE1 can significantly increase nerve agent processing by this serine hydrolase.

### Structural Mechanism for Enhanced Cyclosarin Hydrolysis

**Figure 4:**
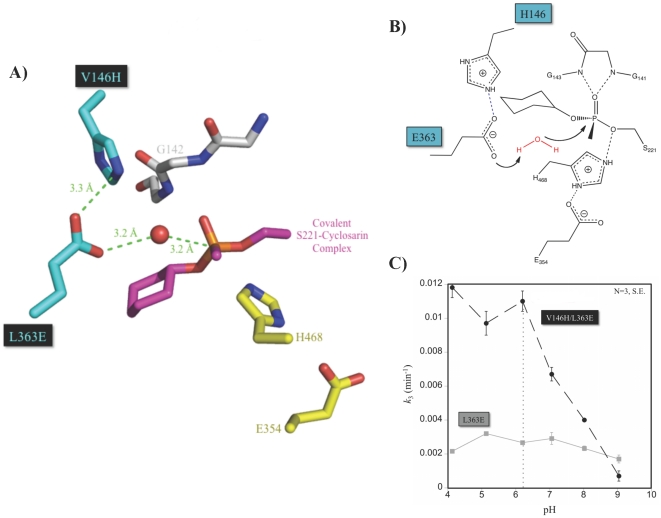
Mechanism of reactivation by V146H/L363E hCE1 after cyclosarin binding. **A**. Model of V146H/L363E (cyan) hCE1 with P*_R_* cyclosarin (magenta) including a water molecule (red) between E363 and the central phosphorus. **B**. Proposed mechanism for enhanced reactivation following cyclosarin inhibition. **C**. pH dependence of V146H/L363E (black) and L363E (grey) hCE1 dephosphonylation following cyclosarin inhibition.

The crystal structure of hCE1 in covalent complex with cyclosarin was then used to generate an energy-minimized model of V146H/L363E hCE1. In the resultant model, the side chains of L363E and V146H were found to be 3.3 Å apart. In addition, the orientation of L363E suggested that a water molecule could be positioned 3.2 Å from L363E and 3.2 Å from the phosphorous atom on the OP-enzyme adduct (**Figure 4A**). Based on this model and the biochemical data presented above, we propose that L363E is positioned for water activation and is stabilized by H146 (**Figure 4B**). Indeed, pH rate dependence studies showed that reactivation proceeded most effectively below pH 6.2, when V146H is likely to be positively charged (**Figure 4C**).

### Bimolecular Rates of Inhibition

To ensure that the enhanced rates of reactivation of V146H/L363E were not artifacts of altered substrate binding, we determined the bimolecular rates of inhibition (*k*_i_) of the mutant protein with racemic cyclosarin or stereoisomers of OP model compounds similar in structure to cyclosarin, and compared those rates to wt enzyme. V146H/L363E hCE1 exhibited a similar *k*_i_ to wt hCE1, confirming that the ability of the mutant protein to bind cyclosarin and related OP compounds was not disrupted (**Table 2** **,** **Table 3**). Indeed, as measured with stereoisomers of the model compounds, V146H/L363E exhibited an improved binding constant (*K*_m_) compared to wt hCE1 with either stereospecific compound [8]. Therefore, the addition of an ion pair across the active site entrance was not detrimental to cyclosarin binding.

**Table 2:** Bimolecular rates of inhibition, Michaelis-Menten constants, and rates of reactivation for wild-type and V146H/L363E hCE1 against racemic cyclosarin and stereoisomers of cyclosarin model compounds.

V146H/L363E	*k*_i_ (×10^3^ M^−1^s^−1^)	*K*_m_ (×10^−6^ M)	*k*_3_ (×10^−4^ min^−1^)
Racemic^a^	79±2	N.D.	67±6
P*_R_* b	5.4±0.4	0.095±0.006	50±10
P*_S_* b	0.12±0.02	5.2±0.7	40±10

WT			
Racemic^a^	11±5	N.D.	<0.01^c^
P*_R_* b	15±1^c^	0.32±0.01^c^	<0.01^c^
P*_S_* b	0.0053±0.0007^c^	1700±200^c^	<0.01^c^

N = 3, s.d., N.D. is not determined, N.R. is no reactivation, pH 7.4, 25°C.

a Racemic *bona fide* cyclosarin,

b stereoisomers of cyclosarin model compounds,

c (8).

**Table 3:** Inhibition and Michaelis-Menten constants for wild-type and V146H/L363E hCE1 against stereoisomers of sarin and soman model compounds.

hCE1	Model Compound	*k*_i_ (×10^3^ M^−1^s^−1^)		*K*_m_ (×10^−6^ M)	
		P*_R_*	P*_S_*	P*_R_*	P*_S_*
WT^a^	Sarin	0.77±0.05	4.1±0.1	1.4±0.2	0.37±0.02
	Soman	2.5±0.5	0.0015±0.0007	0.22±0.02	800±200
V146H/L363E	Sarin	3.40±0.07	4.7±0.2	3.5±0.8	0.9±0.1
	Soman	10.8±0.4	0.67±0.06	0.101±0.005	8±2

(N = 3, s.d.)

a (7).

## Discussion

OP nerve agents serve as hemisubstrates of the wild-type AChE, BChE, and hCE1 enzymes because these chemicals fail to complete the typical two-step serine hydrolase mechanism. Rather, OP nerve agents produce relatively long-lived covalently phosphonylated active site serines [12], [13]. Histidine (H468 in wt hCE1; see Figure 2B) activates the serine (S221 in hCE1) for nucleophilic attack on the OP phosphonyl center. The resultant pentahedral intermediate is stabilized by hCE1 backbone nitrogen atoms in the oxyanion hole (G142 and G143); the fluoride leaving group dissociates upon collapse of the transition state, resulting in a covalently modified enzyme [14]. The resulting tetrahedral phosphonyl adduct adversely affects the ability of H468 to facilitate base mediated C-O bond cleavage [15], which leaves this residue in a state where it cannot activate the water molecule required for hydrolytic desphosphonylation [16]. Thus, for serine hydrolases like AChE and hCE1 that are inhibited by OP hemisubstrates, the addition of a strong oxime is often required to complete the catalytic cycle and regenerate active enzyme. Current treatments following OP exposure include administration of strongly-nucleophilic oximes to dephosphonylate AChE. However, such compounds, such as 2-pralidoxime obidoxime, do not offer broad-spectrum protection against all agents and must be administered quickly [17].

Based on previous crystal structures of hCE1 in covalent complexes with nerve agents [7], [8], we hypothesized that introducing a general base catalyst would facilitate the activation of a properly located water molecule to hydrolyze an OP adduct. The V146H/L363E amino acid substitutions dramatically increased the spontaneous rates of enzyme reactivation following sarin, soman, and cyclosarin inhibition compared to native enzyme (**Figure 3**). A panel of single and double mutant controls indicated that V146H or L363Q facilitated increased sarin hydrolysis, while L363E acted as general base catalyst for cyclosarin and soman hydrolysis. Because V146H, L363Q, or their combination, resulted in similar rates of reactivation, these mutations may enhance dephosphonylation by interacting with the sarin *O*-isopropyl group, thereby affording deprotonation of the catalytic H468 for water activation and hydrolysis. A G117H mutation in BChE resulted in increased rates of dephosphonylation through an analogous mechanism [16], [18].

In contrast, the L363E mutation positions an anionic carboxylate adjacent to the phosphonyl-enzyme intermediate to activate a water molecule for nucleophilic attack. Glutamic acid and aspartic acid residues act as general acid-base catalysts in several established enzyme mechanisms, including those of lysozyme and protein tyrosine phosphatases [19], [20], and apparently support the same role in hCE1. The V146H addition synergistically increased base activity of L363E (**Figure 4A**). The pH profile for cyclosarin hydrolysis by V146H/L363E showed that the fastest rates of hCE1 reactivation was when H146 was likely to be in a charged state, below pH 6.2 and where a cationic H146 can stabilize the anionic base (**Figure 4C**). Based on these data, we postulate that E363 Oε1 is stabilized by H146 and Oε2 on E363 is available to deprotonate a water molecule to hydrolyze the covalent cyclohexyl phosphonyl group (**Figure 4B**). This hypothesis is supported by the observation that the V146Q/L363E variant also exhibited a significant increase in cyclosarin hydrolysis (**Table 1**).

Previous attempts to introduce general base catalysts in OP-inhibited serine hydrolases have resulted in enzymes that also showed enhanced rates of reactivations against soman, sarin, and VX, but the ability of these mutant enzymes to bind the respective OPs was greatly diminished [10], [16]. For example a G117H/E197Q BChE mutant enhanced the rate of soman hydrolysis up to 2,500-fold faster than wt BChE, but the soman bimolecular rate of inhibition for this mutant was decreased by a similar magnitude [10]. In contrast to the G117H/E197Q BChE mutant, V146H/L363E hCE1 exhibits a greater second-order rate of inhibition (*k*_i_) than the wt hCE1 enzyme (**Table 2**). Further, the *K*_m_ values for the cyclosarin model compounds reported in Table 2 demonstrate that the affinity for V146H/L363E hCE1 is greater than the wt enzyme for these analogs. Taken together, these data show that V146H/L363E hCE1 retains greater than wt affinity for cyclosarin, and that the engineered hCE1 mutants enhance rates of reactivation via dephosphonylation rather than decreasing inhibition.

In conclusion, we have rationally designed a variant form of the liver detoxifying enzyme hCE1 that spontaneously dephosphonylates after inhibition by sarin, soman, and cyclosarin up to 33,000-fold faster than wt enzyme. Wild type hCE1 has a catalytic efficiency (*k*_cat_/*K*_m_) [21] towards the standard esterase substrate para-nitrophenylbutyrate (pNPB) of 1.2×10^5^ M^−1^s^−1^ [22]. AChE, one of the most efficient enzymes known, exhibits a *k*_cat_/*K*_m_ close to the diffusion control limit (10^8^ M^−1^s^−1^) for acetylcholine hydrolysis [23]. Combining the nanomolar *K*_m_ of V146H/L363E towards the P*_R_* cyclosarin-like compound along with rate of reactivation after inhibition by this compound, this double mutant shows a catalytic efficiency of 8.8×10^2^ M^−1^s^−1^. Thus, the redesigned hCE1 compares favorably to other mammalian enzymes that have been rationally engineered to improve hemi-substrate metabolism (**Table 4**). This enzyme will likely require substantial increases in catalytic efficiency for OP compounds in order to provide *in vivo* protection, but nonetheless can serve as a lead candidate for further development of novel countermeasures for nerve agent or pesticide poisoning.

**Table 4:** Catalytic efficiencies (*k*_cat_/*K*_m_) of engineered enzymes towards hemisubstrates.

Enzyme	Engineered Activity	Mutation(s)	Catalytic Efficiency (M^−1^s^−1^)
Butyrylcholinesterase	Sarin hydrolysis^a^	G117H	0.79
Glutathione Transferase A1-1	Thioester hydrolysis^b^	A216H	2.60
4-Oxalocrotonate Tautomerase	Oxaloacetate decarboxylase^c^	P1A	114
Phospho(serine/threonine/tyrosine)Binding Protein	Phosphatase activity^c^	G120C	490
**Human Carboxylesterase 1**	**Cyclosarin analog hydrolysis**	**V146H/L363E**	**877**
Cyclophilin	Protease activity^c^	A91S/F104H/N106D	1675

a (16).

b (32).

c (32).

## Materials and Methods

### hCE1 Mutagenesis, Expression, and Purification

Site-directed mutagenesis was done using PCR to introduce the desired amino acid changes into hCE1 (Genbank accession M73499). Briefly, 100 pM of both sense and anti-sense primers were mixed with 50 ng hCE1 cDNA in a pUC9 vector, 200 µM dNTPs, 1× *Pfu*-BSA buffer, and 2 u *Pfu* (New England Biolabs, Ipswich, MA). Mutations were produced through 15 cycles of PCR, where each cycle consisted of 95°C for 1 minute, 58°C for 30 seconds, and 70 C for 10 minutes. The reaction was digested for 1 hour with *Dpn1* (Fermentas, Burlington, Ontario) at 37°C and transformed into chemically competent DH5α cells (Invitrogen, Carlsbad, California). pUC9 plasmids were isolated with a GeneJET Plasmid Miniprep kit (Fermentas) and mutations were confirmed by DNA sequencing. Once the mutations were successfully incorporated, the 1.7 kB hCE1 cDNA was ligated into pCIneo for mammalian cell expression (Promega, Madison, WI). Non-secreted forms of wt and mutant hCE1 proteins were expressed in COS-7 cells (American Type Culture Collection, Rockville, Maryland) as previously described [11]. Presence of active hCE1 was determined by measuring the rate of nitrophenol production in the presence of 3 mM *o*-nitrophenol acetate through a change in absorbance at 420 nm. Specific activities were determined by normalizing hCE1 activity relative to protein expression as visualized through western blot analysis with an anti-hCE1 antibody [11]. To facilitate measurement of additional kinetic constants, secreted forms of wt and V146H/L363E hCE1 were expressed in *Spodoptera frugiperda* Sf21 insect cells (Clontech, Palo Alto, California) using baculovirus-expression vectors and purified as previously described [24].

### Examination of Kinetics of hCE1 Variants

Experiments with all racemic OP nerve agents were conducted at the United States Army Medical Research Institute of Chemical Defense (USAMRICD), Aberdeen Proving Ground, MD. Nerve agents were obtained from the U.S. Army Edgewood Chemical Biological Center (ECBC, Aberdeen Proving Ground, MD). Analysis by nuclear magnetic resonance spectroscopy showed them to be >95% pure. Dilute nerve agent was handled according to safety guidelines in place at USAMRICD. All kinetic assays were conducted at 25°C. In separate experiments, 50 µL of whole cell COS lysates expressing an hCE1 mutant was inhibited with a ∼1000-fold molar excess of racemic sarin, soman, or cyclosarin for 10 minutes. Unbound agent was removed by passing inhibited samples over a CENTRI-SEP size exclusion column (Princeton Separations, Adelphia, NJ). The column eluate was diluted 10-fold in 0.1 M potassium phosphate buffer, pH 7.4. Aliquots were tested for hCE1 carboxylesterase functional activity using 5 mM pNPB and compared to an uninhibited sample. Measurements were taken over 60 hours, and the observed rate of enzyme reactiviation (*k*_obs_), or the ability of enzyme to hydrolyze pNPB, and maximal percent recovery (*A*_max_) were determined by fitting the collected data to equation 1:
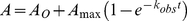
(1)where *A* was the percent activity at time *t*, and *A*_0_ was the initial activity at *t* = 0. Reactivation experiments were conducted in triplicate and independently replicated. It should be noted that these experiments gauge reactivation via a second substrate and do not measure OP turnover or its products directly.

Phosphonylated esterases may either spontaneously reactivate or reside in an inactive state, which is the aged state [8]. However, for wt hCE1 no aging has ever been measured. As shown in **Figure 1B** these two pathways compete as first-order reactions. Therefore, the true rate of enzyme dephosphonylation (*k*_3_) was determined by equation 2 [25]:
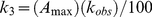
(2)where *k_obs_* and *A*_max_ were defined in eq. 1. The denominator normalizes for *A*_max_. All data were analyzed in KaleidaGraph 4 (Synergy Software, Reading, PA). Because the reactivation step represented by *k*_3_ was the slow step in the OP hydrolysis reaction, *k*_3_ effectively represented the rate of OP turnover.

To determine the bimolecular rate of inhibition (*k*_i_) with racemic cyclosarin, 100 nM of purified V146H/L363E hCE1 or wt enzyme was exposed to 100-fold excess of cyclosarin and the decrease in pNPB hydrolysis was measured over time. Data were fit to equation 3 and the *k*_i_ was calculated by dividing the rate of phosphonylation (*k*_2_) by the cyclosarin concentration:
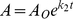
(3)where *A* is the CE activity at a given time *t*, and *A*_0_ is the enzyme activity prior to cyclosarin inhibition.

Further inhibition kinetics were determined with stereoisomers of OP model compounds, where 100 nM of V146H/L363E hCE1 was incubated at room temperature with increasing concentrations of inhibitor. The thiomethylated OPs examined (**Figure 1A**) have previously been shown to form identical adducts to *bona fide* nerve agents [8], [13]. These compounds were generously provided by Dr. John Cashman at the Human BioMolecular Research Institute and their synthesis is described by Gilley et al. [13]. Aliquots of enzyme in the presence of sarin, soman, and cyclosarin model compounds were removed at various time points (up to 1 hour) and the level of remaining enzyme activity was determined by comparing 4-methylumbelliferyl acetate (4-MUA) hydrolysis relative to an uninhibited sample. 4-Methylumbelliferone fluorescence emission, measured at 450 nm following excitation at 350 nm, was monitored at 37°C on a Pherastar microplate reader (BMG Labtech, Cary, NC) and the data were fit to equation 4 [26]:
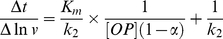
(4)where *K*_m_ was the nerve agent model Michaelis-Menten constant, *k*_2_ the unimolecular phosphonylation rate constant, *v* the remaining percent enzyme activity, and [OP] the OP concentration. The term α was defined as [S]/(*K*_m_+[S]), in which [S] was the substrate concentration and *K*_m_ was the 4-MUA Michaelis-Menten constant. For these experiments α was 0.91. All experiments were performed in triplicate and data were analyzed to determine *k*_i_, where *k*_i_ = *k*_2_/*K*_m_ [16]. Again, all data were analyzed in KaleidaGraph 4.

### Computational Modeling of hCE1 Mutants

To simulate the hCE1-OP crystal structure for computational design, a new residue type was created for S221 covalently inhibited by sarin, soman, or cyclosarin. Following removal of all water molecules, saccharides, ligands, and extra monomers from the hCE1-cyclosarin homotrimer crystal structure (RSCB PDB 3K9B [8]), protein hydrogen atoms were added into one monomer with Molprobity [27]. OP-inhibited S221 hydrogen atoms were added using Accelrys Discovery Studio Visualizer 2.5 (Accelrys, San Diego, CA). Any strain or steric interactions in the resulting structure were then removed by performing 50 steps of conjugate gradient energy minimization.

Residues with side chains pointing towards and within 5 Å of the OP, V146, or L363 residues (using 3K9B numbering, residues 89, 90, 93, 96, 97, 100, 101, 145, 220, 222, 252, 254, 255, 304, 318, 358, 359, 361, 364, 388, 425, 426, and 468,) were allowed to sample alternative conformations during the design phase of the simulation, but their identities were not modified. At positions 146 and 363, only histidine or glutamic acid was allowed, respectively. A standard backbone-dependent side chain rotamer library with expansions by one standard deviation about χ1 and χ2 was used [28]. The crystallographic conformer at each designed position was also allowed. Prior to the modeling procedure, rotamer libraries for the various OPs were generated. The OP rotamer libraries introduced torsions in the phosphorus-alkoxy oxygen and alkoxy oxygen-carbon bonds of each OP-bound serine residue, in 5° increments. The rotamers with conformational energies lower than a specified value (usually 0) were included in the OP rotamer library. Computational design was done using the PHOENIX protein design software. The energy function used was based on the DREIDING force field [29] and included a scaled van der Waals term, hydrogen bonding and electrostatic terms, as well as terms for implicit solvation and phi-psi propensities [29]. Implicit solvation energies were evaluated using a model based on occluded volume. Amino acid phi-psi propensities were derived and applied following the method of Shortle [30]. The final model consisted of the lowest energy rotamers for the OP, V146H, L363E, and surrounding active site residues.
