# Correction: Nerve Agent Hydrolysis Activity Designed into a Human Drug Metabolism Enzyme

**DOI:** 10.1371/annotation/c5953f27-95f8-4a1b-a46c-639d90e182ac

**Published:** 2012-01-23

**Authors:** Andrew C. Hemmert, Tamara C. Otto, Roberto A. Chica, Monika Wierdl, Jonathan S. Edwards, Steven M. Lewis, Carol C. Edwards, Lyudmila Tsurkan, C. Linn Cadieux, Shane A. Kasten, John R. Cashman, Stephen L. Mayo, Philip M. Potter, Douglas M. Cerasoli, Matthew R. Redinbo

There was an error in the sixth author's name. The correct name is Steven M. Lewis. The corrected author contributions are: "Conceived and designed the experiments: ACH RAC MRR. Performed the experiments: ACH TCO RAC MW JSE SML CCE LT CLC SAK. Analyzed the data: ACH RAC JRC SLM PMP DMC MRR. Contributed reagents/materials/analysis tools: ACH TCO RAC MW JSE SML CCE LT CLC SAK. Wrote the paper: ACH TCO RAC MW JSE SML CCE LT CLC SAK JRC SLM PMP DMC MRR." 

